# Graphene Nanocomposites as Innovative Materials for Energy Storage and Conversion—Design and Headways

**DOI:** 10.3390/ijms241411593

**Published:** 2023-07-18

**Authors:** Ayesha Kausar, Ishaq Ahmad, Tingkai Zhao, Osamah Aldaghri, Khalid H. Ibnaouf, M. H. Eisa

**Affiliations:** 1 NPU-NCP Joint International Research Center on Advanced Nanomaterials and Defects Engineering, Northwestern Polytechnical University, Xi’an 710072, China; 2 UNESCO-UNISA Africa Chair in Nanosciences/Nanotechnology, iThemba LABS, Somerset West 7129, South Africa; 3 NPU-NCP Joint International Research Center on Advanced Nanomaterials and Defects Engineering, National Centre for Physics, Islamabad 44000, Pakistan; 4 School of Materials Science & Engineering, Northwestern Polytechnical University, Xi’an 710072, China; 5 Department of Physics, College of Science, Imam Mohammad Ibn Saud Islamic University (IMSIU), Riyadh 13318, Saudi Arabia

**Keywords:** graphene, nanocomposite, polymer, energy storage, conversion, supercapacitor, batteries, fuel cell

## Abstract

This review mainly addresses applications of polymer/graphene nanocomposites in certain significant energy storage and conversion devices such as supercapacitors, Li-ion batteries, and fuel cells. Graphene has achieved an indispensable position among carbon nanomaterials owing to its inimitable structure and features. Graphene and its nanocomposites have been recognized for providing a high surface area, electron conductivity, capacitance, energy density, charge–discharge, cyclic stability, power conversion efficiency, and other advanced features in efficient energy devices. Furthermore, graphene-containing nanocomposites have superior microstructure, mechanical robustness, and heat constancy characteristics. Thus, this state-of-the-art article offers comprehensive coverage on designing, processing, and applying graphene-based nanoarchitectures in high-performance energy storage and conversion devices. Despite the essential features of graphene-derived nanocomposites, several challenges need to be overcome to attain advanced device performance.

## 1. Introduction

Progression in energy technologies demands the use of innovative competent nanomaterials to attain the desired high performance of those technologies [1]. Graphene is one of the most unique nanomaterials adopted for advanced nanocomposite formation [2]. Graphene has the advantages of being lightweight, strong, and eco-friendly, and has superior physical features [3,4]. Moreover, graphene has good electron conductivity and charge-storing properties that are useful for cutting-edge energy and electronic applications like energy production, storage, sensors, electronics, etc. [5,6,7]. The high surface area, electrical, and electrochemical characteristics have been found to be suitable for designing supercapacitor electrodes for charge storage [8]. In addition, graphene has been applied to enhance the charge storage of batteries and fuel cell devices [9]. Supercapacitors with graphene nanomaterials have been used as the most efficient energy storage devices [9]. Moreover, Li-ion batteries employing graphene have been researched for their good energy storage capabilities [10,11]. In addition, graphene-derived materials have also been explored for their use in fuel cells [12]. Utilizing graphene and its related nanomaterials has revealed several valuable features and high performance in terms of charge or energy storing and conversion applications [13]. To design graphene nanomaterials for charge or energy storage and conversion, various facile fabrication methods, matrix–nanofiller interactions, morphology, stability, capacitance, charge density, energy density, cyclic performance, efficiency, and other valued properties have been analyzed [14]. However, to use graphene-derived nanocomposites for high-efficiency energy storage and conversion applications, various design and performance challenges need to be overcome [15,16].

In recent years, functionally graded graphene-reinforced composites have been focused on heavily in the literature [17]. Functionally graded graphene-reinforced composite materials have the advantages of being lightweight and multi-functional and having advanced mechanical and physical features for the development of next-generation devices. The designs of the graphene-reinforced composites have been explored for piezoelectric actuators [18]. Consequently, the influence of a graphene-reinforced composite-based piezoelectric layer has been investigated for these devices. Accordingly, Maxwell’s equation has been used for the piezoelectric layer of the composites. The design of functionally graded graphene-reinforced composites has been found to be valuable for nanoelectromechanical or microelectromechanical systems like nanosensors and nanoactuators. However, defects and imperfections have been observed in functionally graded graphene-reinforced composites [19]. In this context, the 3D poroflexibility theory has been applied to explore the bending responses of the composites. The horizontal friction force and elastic parameters were used for modeling the substrate. The discrete singular convolution integration technique was used to observe the stress–strain responses of the composites. Including higher contents of graphene considerably affected the stress and displacement properties of the composites. Furthermore, the stresses and strains of functionally graded graphene-reinforced composites have been studied using the higher-order shear deformation theory [20]. Here, the module of elasticity was found using the modified Halpin–Tsai model, whereas Poisson’s ratio was utilized to measure the mixture. Hence, the dispersion, conducting, piezoelectric, and mechanical performances of these functional composites have been successfully analyzed using the advanced theoretical and modeling techniques for high-performance energy device applications.

This state-of-the-art review primarily covers the fundamentals of graphene and its nanocomposites. Additionally, the energy storage and conversion solicitations of graphene-derived nanocomposites are scrutinized in terms of important devices. Accordingly, high-efficiency multi-functional supercapacitors, batteries, and fuel cells are debated. To the best of our knowledge, this article is pioneering in the field of energy storage and conversion devices in terms of the review outline, the collected literature, and the wide coverage of various devices to study the effect of graphene-based nanocomposites. Few previous reviews of the literature on graphene materials have included a comprehensive survey in order to support future progress in this field [21]. The future of graphene-derived nanocomposites in various energy storage and conversion systems depends upon overcoming the fabrication- and efficiency-related challenges to form multi-functional supercapacitors, batteries, and fuel cells.

## 2. Graphene: An Exclusive Nanocarbon

**Figure 1:**
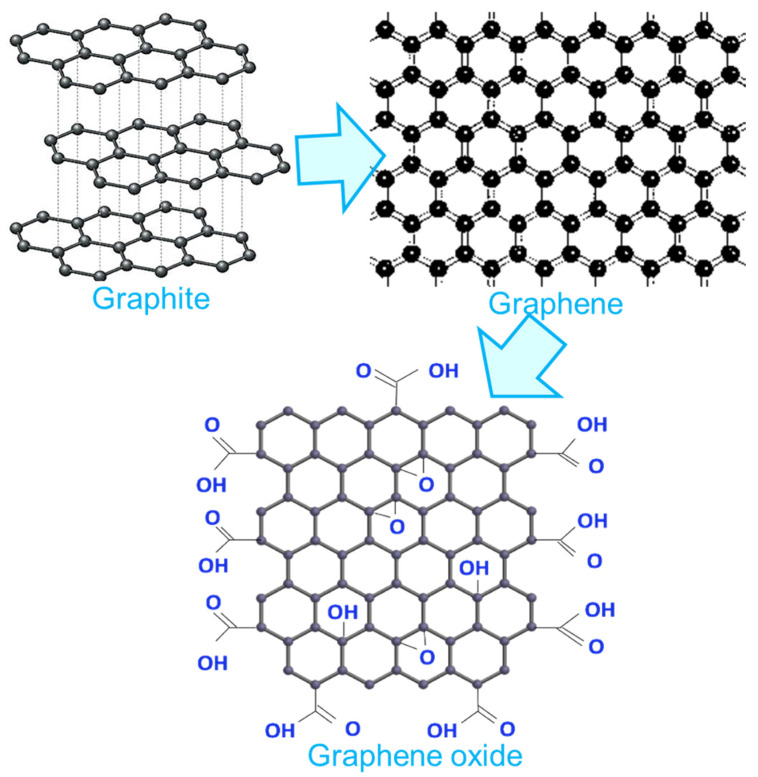
Structure of graphene and related carbon nanomaterials.

Graphene is considered an excellent nanocarbon form [22,23,24]. It has a one-atom-thick nanostructure containing sp^2^-hybridized hexagonal carbon atoms with π-orbitals [25,26,27]. Figure 1 presents the structures of graphene and its interrelated carbon nanomaterials. Initially, graphene was studied by the scientists Geim and Novoselov (Nobel Prize laureates in physics) in 2010 [28,29,30]. Since then, various approaches (top-down or bottom-up) have been adopted to form graphene nanostructures [31,32,33,34]. To name a few, graphite mechanical breakage, exfoliation, chemical vapor deposition, chemical or organic synthesis, and numerous other methodologies have been used [35,36,37,38]. Graphene has a light transparency of 97–98% [39,40]. The electron mobility and thermal conductivity properties of graphene were found to be 200,000 cm^2^V^−1^s^−1^ and 3000–5000 W/mK, respectively [41,42]. In addition, graphene has a very high Young’s modulus of 1 TPa and was found to be 200–300 times more solid relative to steel [43].

**Figure 2:**
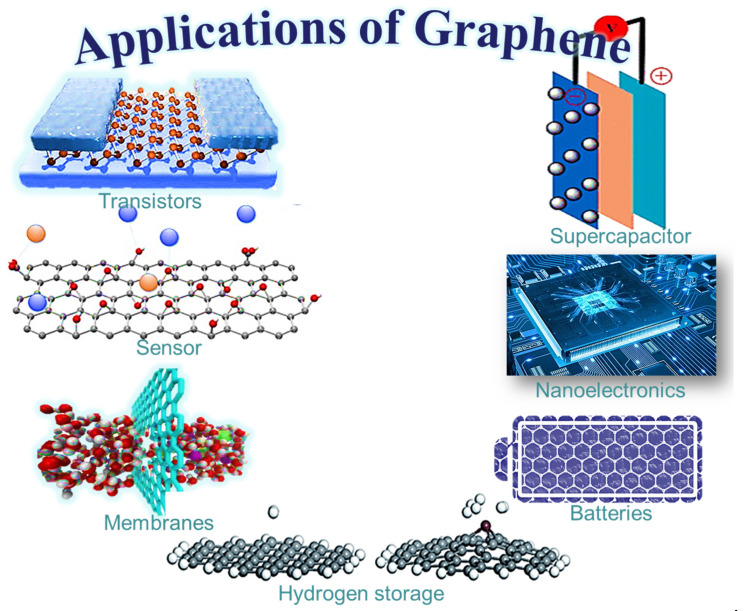
Application areas of graphene.

Graphene nanosheets can be held together through van der Waals interactions [44,45,46]. With all of these remarkable features, graphene and its related materials reveal valuable applications in energy devices and electronics, in the auto or space sector, and in countless other fields [47]. Graphene oxide is developed to be an efficient modified form of graphene, with hydroxyl, carboxylic, epoxide, and several other oxygen-containing surface functionalities and spectacular physical features [48]. Graphene, as well as its derived nanomaterials, have been researched for their superior electrical, thermal, mechanical, and physical features [49,50,51]. Consequently, graphene derivatives and nanomaterials have been used for electronics [52], sensors [53], transistors [54], batteries [55], hydrogen storage [56], and several other devices and applications [57] (Figure 2).

## 3. Graphene-Derived Nanocomposites: Innovative Materials

**Figure 3:**
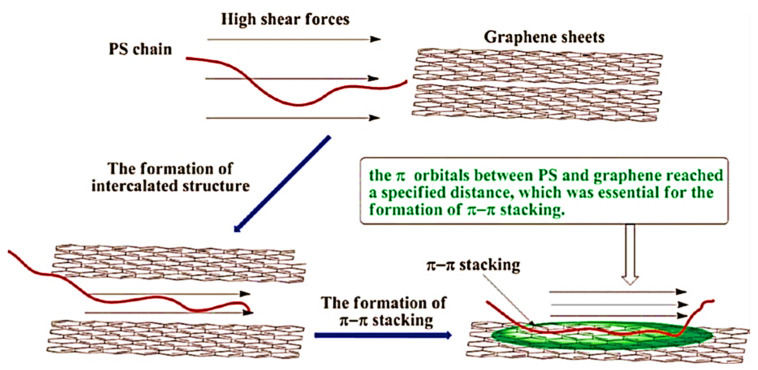
Representation of formation and development of interactions between polymer and graphene [61]. PS = polystyrene. Reproduced with permission from the ACS.

Graphene-derived nanocomposites have been designed and explored for their fabrication, nanofiller contents, nanoparticle scattering, matrix–nanofiller connections, and physical properties [58]. Specifically, polymer/graphene nanocomposites have been developed using various thermoplastics, thermosets, and conjugated polymer matrices. In polyethylene [59] and poly(vinyl alcohol) [60] matrices, the addition of graphene has enhanced the compatibility, electrical conductivity, and strength features of the ensuing nanocomposites. Subsequently, Shen and colleagues [61] designed the polystyrene- and graphene-derived nanocomposites. These nanomaterials depicted π–π interactions between the sp^2^-hybridized structure of graphene and styrene rings of polymer (Figure 3). The aromatic ring associations caused matrix–nanofiller bonding as well as compatibilization. As a result, the heat stability properties of the nanocomposites were found to be enhanced. Zhao and researchers [62] also fabricated polystyrene- and graphene-derived nanocomposites. The interface formation and interactions caused a percolation threshold of 0.0475 vol.% and a high electrical conduction of 20.5 Sm^−1^ [63]. Similarly, polystyrene/graphene nanomaterials were observed to be high in mechanical characteristics due to polymer–filler bonding [64]. In addition, a poly(methyl methacrylate) matrix was also filled with graphene nanofiller [65]. The dispersion of graphene nanoparticles established the interconnected percolation network for electron conduction. Balasubramaniyan and co-workers [66] formed poly(methyl methacrylate)- and graphene-derived nanocomposites. The nanocomposites had an electrical conductivity of 0.039 Sm^−1^. Additionally, the storage modulus and glass transition temperature of the nanocomposites were found to be improved with the addition of graphene. Thus, a graphene additive has been introduced in various polymers to enhance the physical characteristics of these nanomaterials. 

## 4. Graphene Consequent Nanocomposites in Supercapacitors

**Figure 4:**
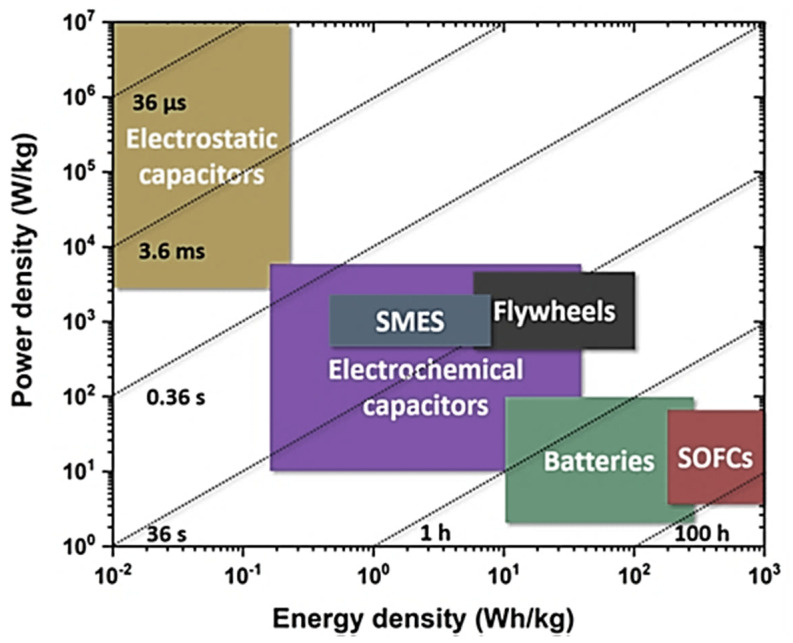
Ragone-plot-based comparative behavior of the numerous energy storage devices studied so far, such as capacitors, superconducting magnetic energy storage capacitors, flywheels, batteries, and solid oxide fuel cells [76]. SMES = superconducting magnetic energy storage; SOFCs = solid oxide fuel cells. Reproduced with permission from Elsevier.

Supercapacitors have been investigated to be notable charge or energy storage devices [67]. Alterations of device structures using efficient nanomaterials may result in enhanced charge-storing capabilities of supercapacitors [68]. Additionally, the use of appropriate processing techniques may improve the device’s performance [69,70,71]. In this context, nanocarbons and polymer/nanocarbon-derived nanomaterials have been used for the synthesis of supercapacitor electrodes due to their high robustness, high charge or power density, as well as capacitance [72,73,74]. Figure 4 reveals the Ragone plots for various energy storage devices. These various charge storage devices were plotted according to their performance. Here, the energy storage devices were developed and analyzed for their efficiency [75]. It can be stated that the capacitors have proven to be the most effectually operative charge storage devices.

**Figure 5:**
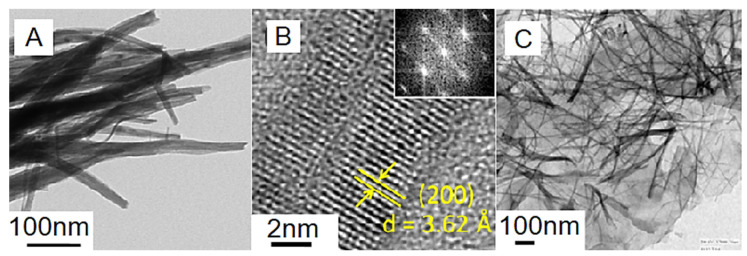
(**A**) TEM image; (**B**) HRTEM image of WO_3_ nanowires (inset is the FFT pattern); and (**C**) TEM images of graphene-WO_3_ nanocomposite [87]. TEM = transmission electron microscope; HRTEM = high resolution transmission electron microscope; FFT = fast Fourier transform. Reproduced with permission from the ACS.

**Figure 6:**
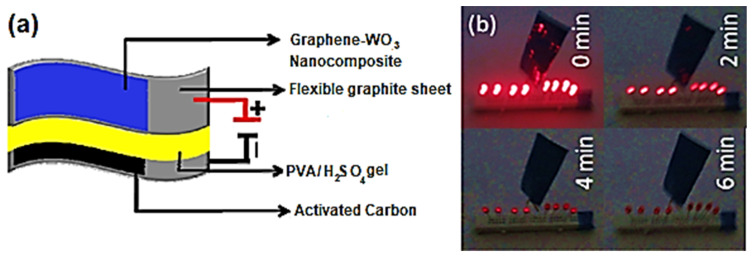
(**a**) Representation of a flexible solid-state asymmetric supercapacitor device and (**b**) diagram of a real ASC device that lights red LEDs after charging [87]. ASC = asymmetric supercapacitor; LED = light emitting diode; PVA = poly(vinyl alcohol). Reproduced with permission from the ACS.

**Figure 7:**
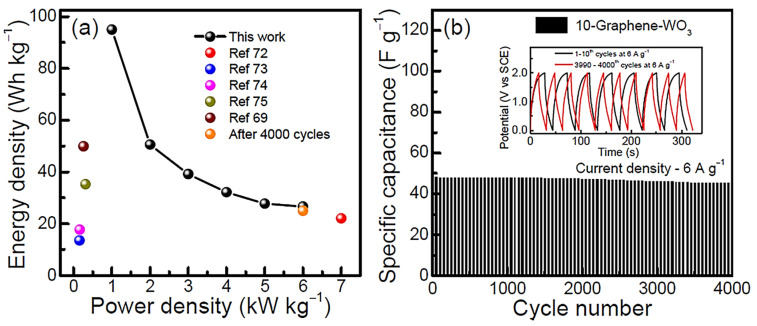
(**a**) Ragone plots of energy density vs. power density and (**b**) cyclic stability plots of specific capacitance as a function of cycle numbers (current density 6.0 A g^−1^) with a two-electrode solid-state ASC cell (graphene-WO_3_ negative electrode and activated carbon positive electrode) [87]. ASC = asymmetric supercapacitor. Reproduced with permission from the ACS.

Graphene has been accredited to be a remarkable carbon nanomaterial [77]. A significant application of graphene was observed for polymeric nanocomposite formation [78,79]. Adding small nanofiller contents to polymer/graphene nanocomposites may increase their desired physical properties [80]. In energy storage devices and systems, graphene and graphene-derived nanocomposites have been effectually useful. A successful design combination for the supercapacitor electrode was developed using conjugated polymers and graphene [81]. Conjugated polymer/graphene nanomaterials have a low cost, structural steadiness, high surface area, capacitance, energy density, and charge/discharge features [82,83,84]. In this context, numerous polymers have been applied to design the supercapacitor electrodes such as polyaniline, polypyrrole, polythiophene, and their derivatives [85,86]. Nayak et al. [87] reported a two-electrode solid-state asymmetric supercapacitor cell derived from graphene-supported tungsten oxide nanowires working as negative electrodes. To check the material performance, the specific capacitance and energy density performance of the supercapacitor were analyzed. Figure 5 demonstrates the transmission electron microscopy and high-resolution transmission electron microscopy images of neat tungsten oxide nanowires and graphene-supported tungsten oxide nanocomposites. Neat tungsten oxide has nanostructures resembling the nanowire-like morphology. In the nanocomposite form, a well-oriented nanostructure was observed owing to the single crystalline nature of the nanomaterial. Figure 6 validates the functioning of a solid-state asymmetric energy storage device. The asymmetric supercapacitor device was applied for lightening the red-colored light-emitting diodes. Furthermore, Figure 7 shows the Ragone plots of the energy density, power density, and cyclic stability profiles of the nanocomposites. Following 4000 charge–discharge cycles, the energy density was maintained in the range of 6 to 25 W h kg^−1^. Henceforth, the graphene nanocomposites revealed superior specific capacitance, energy and power density, and cyclic performance for high-efficiency supercapacitors.

Çıplak and colleagues [88] reinforced the polyaniline matrix with graphene nanofillers, such as graphene oxide and reduced graphene oxide, in addition to the gold nanoparticles. Graphene oxide was developed using the Hummers’ method, whereas the nanocomposite was formed through the in situ polymerization method. The polyaniline/reduced graphene oxide–gold nanoparticle nanocomposite had a specific capacitance of ~213 Fg^−1^, which was 64% higher than the unfilled polyaniline electrode. A superior nanocomposite electrode performance was observed due to the electrostatic and π–π stacking interactions between the conjugated polymer and nanocarbon structures. Arthisree and co-workers [89] fabricated a supercapacitor electrode based on the polyacrylonitrile and polyaniline matrices and graphene quantum dot nanofiller. The electrode had nanofiller contents of up to 1.5 wt.%. The specific capacitance of the nanocomposites was found to be high, in the range of >100 to ~600 Fg^−1^. The superior supercapacitor performance was attributed to the synergistic effects of the conducting polyaniline and polyacrylonitrile as well as the graphene quantum dot additive [90,91]. Thus, the exclusive combinations of polymers and graphene led to high performance designs for advanced supercapacitors.

## 5. Graphene Nanocomposites towards Li-ion Batteries

**Figure 8:**
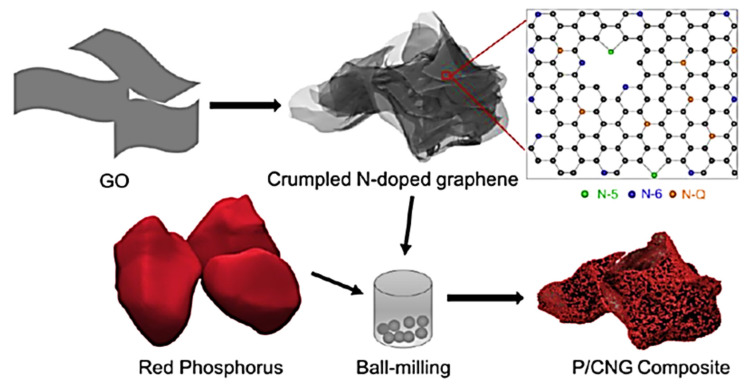
Diagram illustration of the formation of nitrogen-doped graphene and red-phosphorus-derived nanocomposite electrodes [101]. P/CNG = red phosphorus with nitrogen-doped graphene. Reproduced with permission from the ACS.

**Figure 9:**
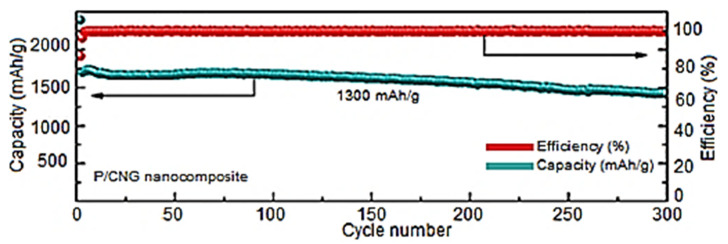
The plots for the capacity vs. cyclic number and efficiency vs. cyclic number for the electrochemical performance of the P/CNG nanocomposite electrode. The long-term cycling behavior of P/CNG nanocomposite electrode can be seen at the current density of 1300 mAhg^−1^ for 300 cycles [101]. P/CNG = red phosphorus with nitrogen-doped graphene. Reproduced with permission from the ACS.

Li-ion batteries have also been used as effective energy storage devices [92,93]. Previously, transition metal-oxide-based electrodes have been applied in these energy devices [94,95]. The continuing research on Li-ion battery electrodes focused on their longevity, large-scale processing, high capacitance, charge or energy storage, and other related properties [96,97]. Consequently, the research has moved towards using graphene nanocomposites and polymer/graphene nanomaterials to attain high specific capacitance and current density (~2000 mAhg^−1^ and >100 mAg^−1^, respectively) features [98]. Li and co-workers [99] designed a Li-ion battery electrode using graphene along with an aligned carbon nanotube. The resulting nanocomposite electrode depicted good capacity and electron conduction as well as robustness. Chang and researchers [100] fabricated a Li-ion battery cathode using graphene- and polysulfur-derived nanocomposites. The homogeneous scattering of graphene in polysulfur established the efficient electron transferring paths in the matrix. Consequently, the battery cathode had a high areal capacity of about 12 mAhcm^−2^. Jiao et al. [101] formed a Li-ion battery anode based on wrinkled nitrogen-doped graphene and red phosphorus. The nitrogen-doped graphene and the red-phosphorus-derived nanocomposite electrodes had a high electron conduction owing to the three-dimensional nature of red phosphorous. Figure 8 shows the formation of the nitrogen-doped graphene and red-phosphorus-derived nanocomposite through a simple and facile ball milling route. On the other hand, the solution route, i.e., Hummers’ method, was used to form the graphene oxide. After that, the cyanamide compound was adopted for the nitrogen doping of the modified graphene to obtain the N-doped graphene. Figure 9 illustrates the electrochemical performance via capacity vs. cyclic number plots and efficiency vs. cyclic number plots. The nitrogen-doped graphene/red-phosphorus-based nanocomposite electrodes revealed a very high reversible discharge capacity of >2000 mAhg^−1^ and an efficiency of ~88% through 100 cycles. Hence, the effectiveness of using graphene in Li-ion battery anodes is established.

Conducting polymer and graphene-derived nanocomposite electrodes have been designed for Li-ion batteries [102]. Li et al. [103] fabricated a polyaniline-grafted graphene-oxide-based battery anode. In these nanomaterials, aromatic π–π linking interactions were observed, which were found to be responsible for a high conductivity and high specific capacity of 900 mAhg^−1^. Moreover, the polyaniline-grafted graphene-oxide-derived anode had a high cycling stability and Coulombic efficiency. Guo and researchers [104] reinforced a conducting polymer poly(4-vinyloxy-2,2,6,6-tetramethyl-piperidine-N-oxyl) using a graphene nanofiller to form the Li-ion battery cathode. The resulting electrode had a high electron conduction, a reasonable specific capacity (270 mAh g^−1^), and an extended cycling life (20,000 cycles). Chae et al. [105] worked on the polyethylenimine- and graphene-oxide-based nanocomposites for the battery electrode. Accordingly, a high reversible capacity of 880 mAhg^−1^ was attained. Hence, the inclusion of graphene and graphene oxide provided considerable benefits to the performance of the Li-ion battery due to dispersion and interactions with the compatible polymer structures [106,107].

In lithium-ion storage batteries, basically different graphene-based nanomaterials have been used for electrodes such as graphene-supported polymers, graphene-supported sulfides, graphene-supported metal oxides or alloys, etc. [108]. The synthesis, morphology, conductivity, electrochemical, and capacitance performances of the graphene-supported nanocomposites need to be focused on for the improvement of lithium-ion storage batteries [109]. An important factor in using graphene nanomaterials in Li-ion batteries is the aggregation prevention for long-time functioning [110]. Thus, graphene materials with a high electrical conductivity must be produced for better battery performance. The production of a three-dimensional porous conductive network may also facilitate the electron transference through the battery materials. The controlled morphologies, satisfactory conductivity, and electrochemical properties as well as the charge storage specification of graphene nanomaterials have been found to be reliant on the microstructure, nanoparticle size, defect number, dispersion, and appropriate nanomaterial composition using conjugated polymer and graphene contents.

## 6. Graphene Nanocomposites for Fuel Cells

**Figure 10:**
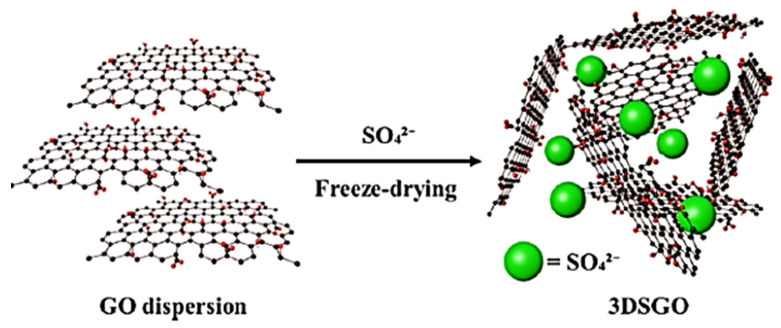
Fabrication of 3DSGO through freeze-drying technique [121]. GO = graphene oxide; 3DSGO = three-dimensional graphene oxide with hydrophilic sulfate ions. Reproduced with permission from the ACS.

**Figure 11:**
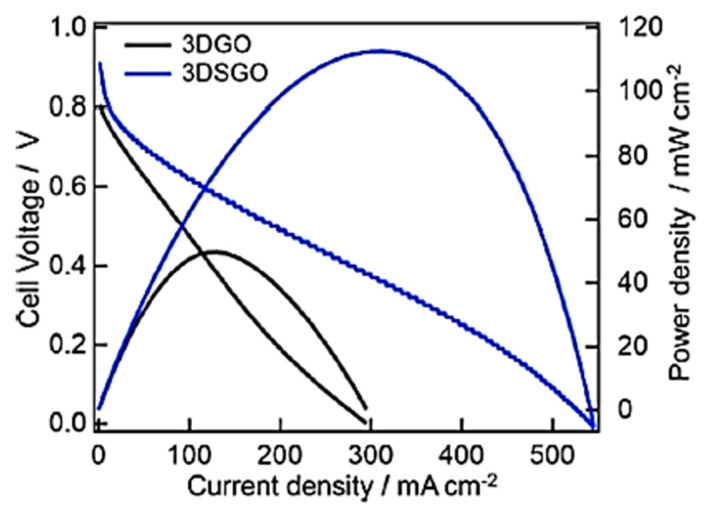
The proton exchange membrane fuel cell performance for 3DGO and 3DSGO membranes functioning at 100% RH (30 °C) [121]. 3DGO = three-dimensional graphene oxide; 3DSGO = three-dimensional graphene oxide with hydrophilic sulfate ions; RH = relative humidity. Reproduced with permission from the ACS.

The fuel cell is an incipient energy conversion technological development employing nanocomposite structures for electrolyte membranes or catalysts [111,112]. Continuous research efforts have been made to develop high-performance fuel cells to improve their electrolyte or catalyst components [113,114,115]. In this context, various nanocarbon nanocomposites have been used in the fuel cell parts [116]. Polypyrrole/graphene oxide nanocomposites have been applied to electrocatalysts and electrode materials [117,118]. The resulting fuel cell revealed a significantly high power conversion efficiency [119,120]. Rahman et al. [121] formed three-dimensional graphene oxide and inserted sulfate ions into the three-dimensional graphene oxide for proton exchange membrane fuel cells. Figure 10 depicts the freeze-drying technique for the insertion of sulfate ions in a three-dimensional graphene oxide nanostructure. The freeze-drying method was found to facilitate the formation of conducting routes as well as the stability of the nanocomposite. Figure 11 presents the fuel cell functioning while using three-dimensional graphene oxide and sulfate ions inserted in three-dimensional graphene oxide nanostructures. The fuel cell was operated at a 100% relative humidity and a temperature of 30 °C. The three-dimensional graphene oxide with inserted sulfate ions had a much higher power density and current density of ~113 mW cm^−2^ and ~311 mA cm^−2^, respectively, relative to the three-dimensional graphene oxide (power density and current density of ~50 mW cm^−2^ and ~121 mA cm^−2^, respectively). Moreover, the modified three-dimensional graphene oxide exhibited an elevated proton conductivity of 3.2 S cm^−1^ relative to the non-modified three-dimensional graphene oxide (0.7 S cm^−1^). The use of the three-dimensional graphene oxide with inserted sulfate ions was suggested to enhance the proton conduction capability of the nanomaterials due to their superior fuel cell performance. 

**Figure 12:**
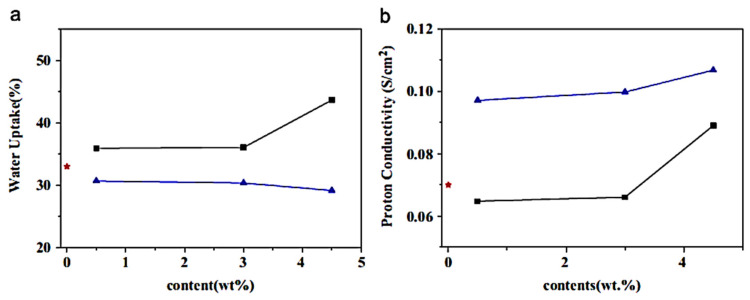
The plots for the (**a**) water uptake and (**b**) proton conductivity of Nafion nanocomposite membranes with (■) GO, (▲) Pt-G, and (*) neat Nafion [122]. GO = graphene oxide; Pt g = platinum–graphene. Reproduced with permission from Elsevier.

Lee et al. [122] fabricated fuel cell membranes based on the neat Nafion, Nafion/graphene oxide, and Nafion/platinum–graphene nanocomposites. Figure 12 displays the water uptake and proton conductivity trends for the pristine Nafion and nanocomposites. Among all systems, the Nafion/graphene oxide revealed a higher water uptake behavior due to the hydrophilicity of graphene oxide. On the other hand, the proton conduction of the Nafion/platinum–graphene nanocomposite was observed to be higher than the neat graphene oxide and the Nafion/graphene oxide nanomaterial. The reason for this is probably the better ion conduction behavior of the interlinked platinum–graphene oxide nanostructure in the polymer matrix. To discover the robustness and stability of the membranes, the mechanical properties were studied (Table 1). Owing to the well-linked nanocomposite formation, the tensile strength of the nanocomposite was found to increase with the graphene oxide loading, whereas the elongation of break gradually decreased. Hence, the high performance Nafion membranes filled with graphene were developed for competent fuel cell applications.

## 7. Challenges and Future

Traditionally designed supercapacitors, batteries, or fuel cells have high price, high density, and low structural reliability problems [123]. Before graphene and its related nanocomposites were adopted, metal and metal-oxide-based electrodes, catalysts, or electrolyte components were greatly used in these energy devices and systems [124]. However, using traditional nanomaterials have several processability, structural, and performance drawbacks towards the functioning of these devices. Here, the designed graphene-based electrodes or other device components reveal remarkable advantages of being low in price, reliable, having a high heat stability, and being environmentally friendly. In addition, using graphene and its derivative nanomaterials may demonstrate high structural stability, superior mechanical robustness, high specific capacitance, high power density, elevated charge density, high charge capacity, superior power conversion efficiency, good cyclic performance, improved recyclability, and a number of other related improved features [125]. 

The commonly used nanocarbon nanofillers for device electrodes and components include graphene, graphite, carbon nanotube, etc. Among these, graphene has been found to be the most efficient to enhance the performance of various energy devices. Moreover, the use of conjugated polymers has been found to enhance the electron and charge transportation through these remarkable nanomaterials [126,127]. It was observed that the inclusion of small amounts of graphene nanomaterials in conjugated polymers notably increased the performance of the energy device components [128]. Additionally, plentiful research efforts have been performed using graphene-nanomaterial-based energy storing or converting components to attain a high surface area, good electrochemical performance, capacitance, charge capacity, charge density, power conversion efficiency, and other characteristics [129,130]. Despite these advantages, graphene-nanomaterial-derived energy device components have some drawbacks. Most importantly, pristine graphene may have low charge storing and energy conversion properties. Therefore, graphene nanomaterials cannot be used without the appropriate structural alterations or nanocomposite formation. The combinations of the graphene nanomaterials, especially modified graphene and conducting polymers, have been suggested in the literature. The functional graphene nanoparticles such as graphene oxide may offer good amalgamation with polymer matrices for various energy components. Furthermore, the electron conduction, supercapacitance, and fuel cell performance of the related electrodes and electrolytes have been found to be dependent upon the combination of conjugated polymers and graphene. Studies on the addition of optimum amounts of graphene or conducting polymers can be carried out to attain high efficiency electrodes in the future. For upcoming developments in this field, comprehensive research efforts on the structure–property relationships of graphene-derived nanocomposites must be performed. 

More specifically, in supercapacitors, different graphene-derived nanocomposites have been utilized. Polymer/graphene nanocomposites with a high surface area and pseudocapacitance may result in a fabulous enhancement in the supercapacitor performance [131]. Consequently, multidisciplinary approaches have been found to be essential to understand the association between microstructure, electrochemistry, materials chemistry/physics, engineering, physical features, and interactions to overcome the significant challenges of these energy storage systems [132]. Improving these lines may resolve the worldwide critical energy issues that are causing global challenges [133]. 

Although promising results have been attained for numerous graphene-nanocomposite-derived Li-ion battery electrode materials, there are some prevailing challenges that need to be overcome for future developments [134]. Accordingly, the thorough understanding of the lithium storage mechanism in graphene-derived nanocomposites has been found to be indispensable to overcome the challenges regarding the surface defects, functionalities, and hierarchical electrode structures. The graphene dispersion in the nanocomposites has been considered essential for better Li-ion storage capacity and facile lithium intercalation [135]. The conductivity and mechanical robustness of graphene nanosheets need to be improved to sustain the repetitive cycling process. The designs of graphene-nanocomposite-derived Li-ion battery electrodes need to be focused on the rapid lithium ion insertion and extraction processes, stable output of energy, and power density of the batteries [136]. In addition, the high cost of graphene-nanocomposite-derived electrodes may significantly limit the scalable production and Li-ion battery application. Moreover, the high surface area of graphene nanomaterials may be advantageous, but also cause large irreversible capacity losses. Similarly, porosity in the nanocomposite may also be unfavorable due to the low volumetric capacity of the Li-ion battery. To achieve a superior electrochemical performance, an understanding the interfacial interactions in graphene nanomaterials, morphology control, porosity, defects, and graphene alignment in the nanocomposites have been found to be essential. The advanced graphene nanocomposite electrodes of Li-ion batteries can be effectively used for the development of future electronics, scalable energy storage devices, and electric or hybrid electric vehicles [137].

The inclusion of graphene in nanocomposites may cause high performance and resilience for fuel-cell-based energy conversion devices [138]. Graphene nanocomposites have been used in the electrodes, bipolar plates, and proton-conducting membranes of fuel cells. In electrodes, a high electrochemically active surface area is desirable for better electro-catalytic activity through fuel oxidation–reduction reactions. In the bipolar plates of fuel cells, durability, anticorrosion, and high performance must be achieved by appropriately incorporating the dispersed graphene nanomaterials [139]. In proton exchange membranes, graphene nanocomposite electrolytes must have good ionic conductivity, power density, and membrane performance [140]. Thus, perfectly designed graphene-based fuel cell devices with all controlled factors may have a worthy performance for the upcoming advanced commercial applications.

**Figure 13:**
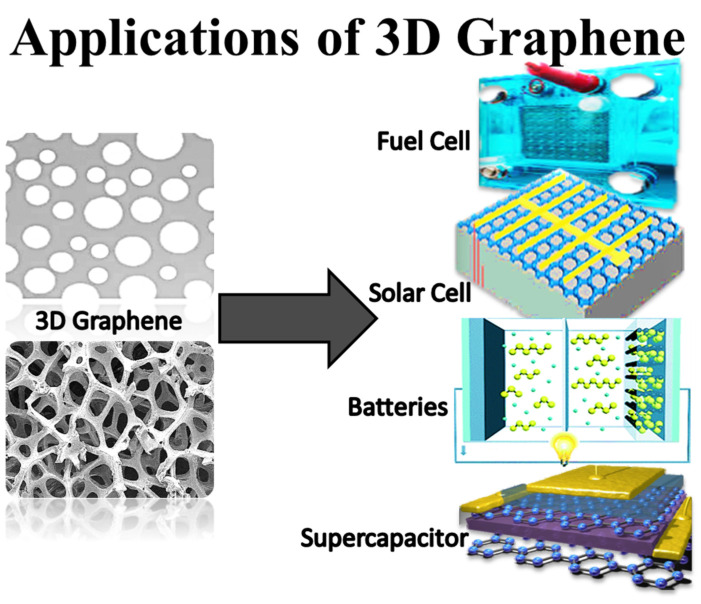
The uses of three-dimensional graphene in energy systems.

Henceforward, the future of graphene nanomaterials in energy devices greatly rely on the development of new innovative materials. In this context, using three-dimensional graphene-derived nanocomposites in energy systems may bring revolution in this field (Figure 13). The conversion of two-dimensional graphene into a three-dimensional network has revealed exceptional features for high-tech applications. The enormous progress in the field of energy devices has led to the utilization of three-dimensional graphene architectures [141]. However, there is an immense need for developing three-dimensional graphene-derived nanocomposite materials to expand the energy-related potential of graphene nanofoams.

Facile approaches need to be adopted to achieve efficient graphene-derived nanocomposites [142]. High-performance graphene nanomaterials have a range of superior physical characteristics including morphological properties, electron conduction, heat constancy, mechanical stability, reliability, and so on. All of these properties of graphene-derived nanomaterials rely on good interactions as well as compatibility between nanocomposite components, i.e., the polymer and nano-reinforcement [143,144]. As various structural combinations of graphene, graphene derivatives, and conducting or non-conducting polymers have been designed for high-end devices, enhancing their conductivity or capacitance may sometimes reduce the structural stability of the nanomaterials [145]. Consequently, the design of graphene nanocomposites must be carefully focused for high-efficiency devices and systems. Fewer studies have considered the actual mechanisms behind the charge storage and energy conversion or production capabilities of these devices, and so comprehensive research efforts were found to be necessary in this field. Accordingly, the matrix–nanofiller interface and matrix–nanofiller interactions (hydrogen bonding, electrostatic interactions, covalent links, etc.) must be considered along with the consistent nanoparticle dispersion in the ensuing nanomaterials. Hence, plenty of future focused investigations on emerging energy device components based on graphene nanomaterials are thought to be necessary [146]. In addition to energy devices, the high-tech future of graphene nanocomposites can be observed in electronics, microwaves, telecommunication, and interrelated devices.

## 8. Summary

This overview exclusively and comprehensively debates the progresses in the arena of graphene and graphene-derived nanocomposites, concentrating on the energy storage and conversion solicitations. Graphene-derived nanomaterials have been constantly researched for supercapacitance, Li-ion batteries, and fuel cell applications. These devices have been categorized among the most essential energy storage and conversion devices. The investigations exposed the stability, charge storage, charge capacity, and conversion efficiency of these energy systems while employing some efficient carbon nanomaterials. In the case of advanced supercapacitors and batteries, using graphene, graphene oxide, functional graphene, and related nanocomposite nanomaterials amended the charge storage performance as well as the power conversion and utilization capabilities of the devices. The employed polymers along with the graphene-based nanofillers were found to be important to develop the high-efficiency energy-associated devices and systems. In other words, the choice of graphene nano-reinforcements along with the selection of polymers were considered indispensable for a better performance of the energy devices. In addition, the graphene modification as well as the graphene nanocomposite processing methods have played imperative roles in the enhancement of the charge storage and energy-production-related performance of these materials, resulting in high-efficacy supercapacitors and other devices. Consequently, the indispensable features of the energy devices and systems were enhanced using graphene or its derived nanofillers, graphene synthesis methods, nanocomposite fabrication techniques, etc., leading to homogeneous microstructure, matrix–graphene interactions, electron conduction, specific capacitance, charge capacity, high charge storing capability, charge–discharge, life cycle, charge density, power density, cyclic concert, reliability, and numerous related features of these systems.

Briefly speaking, in this article, we debated the topical research advancements in the graphene and graphene-derived nanocomposites for important energy storage and conversion systems. Although substantial developments have been made so far, the marvelous potential for real-world applications in cutting-edge energy systems still requires further research. Importantly, the fabrication of graphene and its related nanocomposites for diverse energy applications are still in premature stages, where gaps exist in understanding the atomic/molecular level functioning, essential limits, and failure of devices. Moreover, the high surface area of graphene-derived nanomaterials plays a significant part in supercapacitors and batteries for charge storage. The overall nanocomposite design supporting the combination of a high surface area and pseudocapacitance of graphene may result in a tremendous improvement in the charge-storing potential of devices. To overcome the challenges of energy storage systems, it is essential to develop and understand the multidisciplinary tactics and connections between materials science, engineering, microstructures, electrochemistry, physical features, and interactions.

Research has revealed the potential of graphene nanocomposites towards the electrodes of rechargeable Li-ion batteries. The high-energy densities of Li-based materials were found to be favorable for electronic energy storage. Mostly, the anode systems in Li-ion batteries benefited from using graphene nanocomposites due to their high surface area and conductivity values. All of these factors caused the high power density and rate performance of the Li-ion battery anode. In this context, the high surface area of graphene can accommodate more Li-ions to improve the energy density and capacity of the batteries. Carefully designed graphene-nanocomposite-based electrodes have been applied for commercial-level applications. On the other hand, the high surface area, aggregation trends, and porosity of graphene-derived nanomaterials can also decrease the performance of the electrode materials due to the formation of the solid electrolyte interphase layer causing a low first cycle Coulombic efficiency. Hence, the overall practical applications of graphene nanocomposites in energy storage systems have been limited. Moreover, using porous graphene-based materials may absorb large amounts of electrolytes, leading to battery swelling during electrochemical processes and also cost issues.

Moreover, unusually high physical/chemical properties of graphene nanocomposites have been used for fuel cell applications due to their efficient proton conduction. Graphene-nanocomposite-derived fuel cell catalysts have cost effectiveness, high durability, and insensitivity to carbon monoxide during electrocatalysis relative to platinum-based catalysts. In proton-conducting electrolytes, graphene derivatives have oxygen functionalities to hold water and develop the proton transportation channels. However, due to blending with polymers, some challenges may arise like aggregation, decreased surface area, and homogeneity of the electrolyte membranes. Hence, graphene has been blended with different polymers in order to fabricate nanocomposites for enhancing the processing, mechanical stability, chemical and electrochemical features, and other functions for applications in supercapacitors, batteries, and fuel cells. Although great evolutions have already been attained, the research in this field still faces several unresolved glitches.

Hence, this overview portrays the developments in the field of graphene nanomaterials for energy storage and conversion. In this context, several graphene nanofillers and derived nanomaterials along with polymers such as conjugated polymers, thermoplastics, and thermosets have been used for nanocomposites in order to attain advanced features of the energy devices. Appropriately adopting graphene-derived nanomaterials offers some notable future opportunities towards important energy storage and conversion systems. Moreover, constant research efforts may resolve the challenges related to the design, properties, and performance of applying graphene materials in energy devices. Forthcoming research may extend the use of the above conversed graphene-based nanomaterials towards efficient photovoltaics, light-emitting diodes, nanogenerators, microelectronics, and numerous other devices.

## Figures and Tables

**Table 1:** The tensile strength and elongation at break features of casting Nafion and Nafion/GO nanocomposite membranes [122]. GO = graphene oxide. Reproduced with permission from Elsevier.

Sample	Tensile Strength (MPa)	Elongation at Break (%)
Nafion	9.41	88.30
Nafion/GO 0.5	65.16	31.85
Nafion/GO 3.0	74.69	22.68
Nafion/GO 4.5	79.47	19.12

## References

[1] Fraenza C.C., Greembaum S., Suarez S.N. (2023). Nuclear Magnetic Resonance Relaxation Pathways in Electrolytes for Energy Storage. Int. J. Mol. Sci..

[2] Tero R., Hagiwara Y., Saito S. (2023). Domain Localization by Graphene Oxide in Supported Lipid Bilayers. Int. J. Mol. Sci..

[3] Park L., Kim H.-S., Jang W., Ji M.-K., Ryu J.-H., Cho H., Lim H.-P. (2023). Antibacterial Evaluation of Zirconia Coated with Plasma-Based Graphene Oxide with Photothermal Properties. Int. J. Mol. Sci..

[4] Polyakova P.V., Baimova J.A. (2023). Mechanical Properties of Graphene Networks under Compression: A Molecular Dynamics Simulation. Int. J. Mol. Sci..

[5] Kausar A., Ahmad I., Eisa M., Maaza M., Khan H. (2023). Manufacturing Strategies for Graphene Derivative Nanocomposites—Current Status and Fruitions. Nanomanufacturing.

[6] Adnan M. (2023). The Future of Energy Storage: Advancements and Roadmaps for Lithium-Ion Batteries. Int. J. Mol. Sci..

[7] Wang F., Han Y., Feng X., Xu R., Li A., Wang T., Deng M., Tong C., Li J., Wei Z. (2023). Mesoporous Carbon-Based Materials for Enhancing the Performance of Lithium-Sulfur Batteries. Int. J. Mol. Sci..

[8] Chen G.Z. (2017). Supercapacitor and supercapattery as emerging electrochemical energy stores. Int. Mater. Rev..

[9] Smaisim G.F., Abed A.M., Al-Madhhachi H., Hadrawi S.K., Al-Khateeb H.M.M., Kianfar E. (2023). Graphene-based important carbon structures and nanomaterials for energy storage applications as chemical capacitors and supercapacitor electrodes: A review. BioNanoScience.

[10] Fallahifar R., Kalantar M. (2023). Optimal planning of lithium ion battery energy storage for microgrid applications: Considering capacity degradation. J. Energy Storage.

[11] Sengodu P., Deshmukh A.D. (2015). Conducting polymers and their inorganic composites for advanced Li-ion batteries: A review. Rsc Adv..

[12] Wang X., Duan L., Zheng N. (2023). Thermodynamic analysis of a novel tri-generation system integrated with a solar energy storage and solid oxide fuel cell–Gas turbine. Appl. Therm. Eng..

[13] Ahmadi Y., Ahmad S. (2019). Surface-active antimicrobial and anticorrosive Oleo-Polyurethane/graphene oxide nanocomposite coatings: Synergistic effects of in-situ polymerization and π-π interaction. Prog. Org. Coat..

[14] Mohan T., Kanny K. (2020). Green Nanofillers for Polymeric Materials. Green Nanomaterials.

[15] Siwal S.S., Zhang Q., Devi N., Thakur V.K. (2020). Carbon-based polymer nanocomposite for high-performance energy storage applications. Polymers.

[16] Mooney M., Nyayachavadi A., Rondeau-Gagné S. (2020). Eco-friendly semiconducting polymers: From greener synthesis to greener processability. J. Mater. Chem. C.

[17] Zhao S., Zhao Z., Yang Z., Ke L., Kitipornchai S., Yang J. (2020). Functionally graded graphene reinforced composite structures: A review. Eng. Struct..

[18] Ghabussi A., Ashrafi N., Shavalipour A., Hosseinpour A., Habibi M., Moayedi H., Babaei B., Safarpour H. (2021). Free vibration analysis of an electro-elastic GPLRC cylindrical shell surrounded by viscoelastic foundation using modified length-couple stress parameter. Mech. Based Des. Struct. Mach..

[19] Huo J., Zhang G., Ghabussi A., Habibi M. (2021). Bending analysis of FG-GPLRC axisymmetric circular/annular sector plates by considering elastic foundation and horizontal friction force using 3D-poroelasticity theory. Compos. Struct..

[20] Ghabussi A., Habibi M., NoormohammadiArani O., Shavalipour A., Moayedi H., Safarpour H. (2021). Frequency characteristics of a viscoelastic graphene nanoplatelet–reinforced composite circular microplate. J. Vib. Control.

[21] Yang H. A review of supercapacitor-based energy storage systems for microgrid applications. In Proceedings of 2018 IEEE Power & Energy Society General Meeting (PESGM).

[22] Novoselov K.S., Fal V., Colombo L., Gellert P., Schwab M., Kim K. (2012). A roadmap for graphene. Nature.

[23] Usachov D., Adamchuk V., Haberer D., Grüneis A., Sachdev H., Preobrajenski A., Laubschat C., Vyalikh D. (2010). Quasifreestanding single-layer hexagonal boron nitride as a substrate for graphene synthesis. Phys. Rev. B.

[24] Gao Y., Zhang Y., Chen P., Li Y., Liu M., Gao T., Ma D., Chen Y., Cheng Z., Qiu X. (2013). Toward single-layer uniform hexagonal boron nitride–graphene patchworks with zigzag linking edges. Nano Lett..

[25] Girit Ç.Ö., Meyer J.C., Erni R., Rossell M.D., Kisielowski C., Yang L., Park C.-H., Crommie M., Cohen M.L., Louie S.G. (2009). Graphene at the edge: Stability and dynamics. Science.

[26] Gómez-Navarro C., Meyer J.C., Sundaram R.S., Chuvilin A., Kurasch S., Burghard M., Kern K., Kaiser U. (2010). Atomic structure of reduced graphene oxide. Nano Lett..

[27] Huang P.Y., Ruiz-Vargas C.S., Van Der Zande A.M., Whitney W.S., Levendorf M.P., Kevek J.W., Garg S., Alden J.S., Hustedt C.J., Zhu Y. (2011). Grains and grain boundaries in single-layer graphene atomic patchwork quilts. Nature.

[28] Geim A.K., Novoselov K.S. (2007). The rise of graphene. Nat. Mater..

[29] Geim A.K. (2011). Nobel Lecture: Random walk to graphene. Rev. Mod. Phys..

[30] Novoselov K.S., Geim A.K., Morozov S.V., Jiang D.-E., Zhang Y., Dubonos S.V., Grigorieva I.V., Firsov A.A. (2004). Electric field effect in atomically thin carbon films. Science.

[31] Avouris P., Dimitrakopoulos C. (2012). Graphene: Synthesis and applications. Mater. Today.

[32] Tour J.M. (2014). Top-down versus bottom-up fabrication of graphene-based electronics. Chem. Mater..

[33] Huang Y.-H., Bao Q., Duh J.-G., Chang C.-T. (2016). Top-down dispersion meets bottom-up synthesis: Merging ultranano silicon and graphene nanosheets for superior hybrid anodes for lithium-ion batteries. J. Mater. Chem. A.

[34] Zhang Z., Fraser A., Ye S., Merle G., Barralet J. (2019). Top-down bottom-up graphene synthesis. Nano Futures.

[35] Wei C., Negishi R., Ogawa Y., Akabori M., Taniyasu Y., Kobayashi Y. (2019). Turbostratic multilayer graphene synthesis on CVD graphene template toward improving electrical performance. Jpn. J. Appl. Phys..

[36] Cabrero-Vilatela A., Weatherup R.S., Braeuninger-Weimer P., Caneva S., Hofmann S. (2016). Towards a general growth model for graphene CVD on transition metal catalysts. Nanoscale.

[37] Kim K.S., Zhao Y., Jang H., Lee S.Y., Kim J.M., Kim K.S., Ahn J.-H., Kim P., Choi J.-Y., Hong B.H. (2009). Large-scale pattern growth of graphene films for stretchable transparent electrodes. Nature.

[38] Wang M., Jang S.K., Jang W.J., Kim M., Park S.Y., Kim S.W., Kahng S.J., Choi J.Y., Ruoff R.S., Song Y.J. (2013). A Platform for Large-Scale Graphene Electronics–CVD Growth of Single-Layer Graphene on CVD-Grown Hexagonal Boron Nitride. Adv. Mater..

[39] Liang J., Li L., Tong K., Ren Z., Hu W., Niu X., Chen Y., Pei Q. (2014). Silver nanowire percolation network soldered with graphene oxide at room temperature and its application for fully stretchable polymer light-emitting diodes. ACS Nano.

[40] Narayanam P.K., Botcha V.D., Ghosh M., Major S.S. (2019). Growth and Photocatalytic Behaviour of Transparent Reduced GO-ZnO Nanocomposite Sheets. Nanotechnology.

[41] Kausar A. (2018). Potential of polymer/graphene nanocomposite in electronics. Am. J. Nanosci. Nanotechnol. Res..

[42] Hu K., Kulkarni D.D., Choi I., Tsukruk V.V. (2014). Graphene-polymer nanocomposites for structural and functional applications. Prog. Polym. Sci..

[43] Zandiatashbar A., Lee G.-H., An S.J., Lee S., Mathew N., Terrones M., Hayashi T., Picu C.R., Hone J., Koratkar N. (2014). Effect of defects on the intrinsic strength and stiffness of graphene. Nat. Commun..

[44] Yang S.-T., Chang Y., Wang H., Liu G., Chen S., Wang Y., Liu Y., Cao A. (2010). Folding/aggregation of graphene oxide and its application in Cu2+ removal. J. Colloid Interface Sci..

[45] Wang W.-N., Jiang Y., Biswas P. (2012). Evaporation-induced crumpling of graphene oxide nanosheets in aerosolized droplets: Confinement force relationship. J. Phys. Chem. Lett..

[46] Zhou Q., Xia G., Du M., Lu Y., Xu H. (2019). Scotch-tape-like exfoliation effect of graphene quantum dots for efficient preparation of graphene nanosheets in water. Appl. Surf. Sci..

[47] Mohan V.B., Lau K.-t., Hui D., Bhattacharyya D. (2018). Graphene-based materials and their composites: A review on production, applications and product limitations. Compos. Part B Eng..

[48] Pei S., Cheng H.-M. (2012). The reduction of graphene oxide. Carbon.

[49] Lee H., Lee K.S. (2019). Interlayer Distance Controlled Graphene, Supercapacitor and Method of Producing the Same. U.S. Patent.

[50] Ke Q., Wang J. (2016). Graphene-based materials for supercapacitor electrodes—A review. J. Mater..

[51] Shen X.J., Zeng X.L., Dang C.Y. (2019). Graphene Composites. Handb. Graphene.

[52] Han J.T., Jang J.I., Cho J.Y., Hwang J.Y., Woo J.S., Jeong H.J., Jeong S.Y., Seo S.H., Lee G.-W. (2017). Synthesis of nanobelt-like 1-dimensional silver/nanocarbon hybrid materials for flexible and wearable electronics. Sci. Rep..

[53] Panwar N., Soehartono A.M., Chan K.K., Zeng S., Xu G., Qu J., Coquet P., Yong K.-T., Chen X. (2019). Nanocarbons for biology and medicine: Sensing, imaging, and drug delivery. Chem. Rev..

[54] Romagnoli A., D’Agostino M., Pavoni E., Ardiccioni C., Motta S., Crippa P., Biagetti G., Notarstefano V., Rexha J., Perta N. (2023). SARS-CoV-2 multi-variant rapid detector based on graphene transistor functionalized with an engineered dimeric ACE2 receptor. Nano Today.

[55] Bi J., Du Z., Sun J., Liu Y., Wang K., Du H., Ai W., Huang W. (2023). On the Road to the Frontiers of Lithium-ion Batteries: A Review and Outlook of Graphene Anodes. Adv. Mater..

[56] Macili A., Vlamidis Y., Pfusterschmied G., Leitgeb M., Schmid U., Heun S., Veronesi S. (2023). Study of hydrogen absorption in a novel three-dimensional graphene structure: Towards hydrogen storage applications. Appl. Surf. Sci..

[57] Tang C., Titirici M.-M., Zhang Q. (2017). A review of nanocarbons in energy electrocatalysis: Multifunctional substrates and highly active sites. J. Energy Chem..

[58] Ganesan V., Jayaraman A. (2014). Theory and simulation studies of effective interactions, phase behavior and morphology in polymer nanocomposites. Soft Matter.

[59] Kuila T., Bose S., Hong C.E., Uddin M.E., Khanra P., Kim N.H., Lee J.H. (2011). Preparation of functionalized graphene/linear low density polyethylene composites by a solution mixing method. Carbon.

[60] Vadukumpully S., Paul J., Mahanta N., Valiyaveettil S. (2011). Flexible conductive graphene/poly (vinyl chloride) composite thin films with high mechanical strength and thermal stability. Carbon.

[61] Shen B., Zhai W., Chen C., Lu D., Wang J., Zheng W. (2011). Melt blending in situ enhances the interaction between polystyrene and graphene through π–π stacking. ACS Appl. Mater. Interfaces.

[62] Zhao F., Zhang G., Zhao S., Cui J., Gao A., Yan Y. (2018). Fabrication of pristine graphene-based conductive polystyrene composites towards high performance and light-weight. Compos. Sci. Technol..

[63] He F., Lam K.-H., Fan J., Chan L.H. (2014). Improved dielectric properties for chemically functionalized exfoliated graphite nanoplates/syndiotactic polystyrene composites prepared by a solution-blending method. Carbon.

[64] Mohammadsalih Z.G., Inkson B.J., Chen B. (2021). The effect of dispersion condition on the structure and properties of polystyrene/graphene oxide nanocomposites. Polym. Compos..

[65] Zeng X., Yang J., Yuan W. (2012). Preparation of a poly (methyl methacrylate)-reduced graphene oxide composite with enhanced properties by a solution blending method. Eur. Polym. J..

[66] Balasubramaniyan R., Pham V.H., Jang J., Hur S.H., Chung J.S. (2013). A one pot solution blending method for highly conductive poly (methyl methacrylate)-highly reduced graphene nanocomposites. Electron. Mater. Lett..

[67] Ma N., Yang D., Riaz S., Wang L., Wang K. (2023). Aging Mechanism and Models of Supercapacitors: A Review. Technologies.

[68] Liu S., Yu T., Wu Y., Li W., Li B. (2014). Evolution of cellulose into flexible conductive green electronics: A smart strategy to fabricate sustainable electrodes for supercapacitors. RSC Adv..

[69] Alsaad A.M., Aljarrah I.A., Ahmad A., Al-Bataineh Q.M., Shariah A., Al-Akhras M.A., Telfah A.D. (2022). The structural, optical, thermal, and electrical properties of synthesized PEO/GO thin films. Appl. Phys. A.

[70] Singh J., Dhaliwal A., Sharma K., Sehgal R., Kumar V. (2022). Conductive polymer-based composite photocatalysts for environment and energy applications. Conjugated Polymers for Next-Generation Applications.

[71] Albarqouni Y.M., Lee S.P., Ali G.A., Ethiraj A.S., Algarni H., Chong K.F. (2022). Facile synthesis of reduced graphene oxide aerogel in soft drink as supercapacitor electrode. J. Nanostruct. Chem..

[72] Borenstein A., Hanna O., Attias R., Luski S., Brousse T., Aurbach D. (2017). Carbon-based composite materials for supercapacitor electrodes: A review. J. Mater. Chem. A.

[73] Iro Z.S., Subramani C., Dash S. (2016). A brief review on electrode materials for supercapacitor. Int. J. Electrochem. Sci.

[74] Liu X., Li K. (2020). Energy storage devices in electrified railway systems: A review. Transp. Saf. Environ..

[75] Yao K., Chen S., Rahimabady M., Mirshekarloo M.S., Yu S., Tay F.E.H., Sritharan T., Lu L. (2011). Nonlinear dielectric thin films for high-power electric storage with energy density comparable with electrochemical supercapacitors. IEEE Trans. Ultrason. Ferroelectr. Freq. Control.

[76] Yang L., Kong X., Li F., Hao H., Cheng Z., Liu H., Li J.-F., Zhang S. (2019). Perovskite lead-free dielectrics for energy storage applications. Prog. Mater. Sci..

[77] Kausar A. (2023). N-Doped Graphene and Polymer Sequent Nanocomposite—Nitty-Gritties and Scoping Insights. Polym. Plast. Technol. Mater..

[78] Kausar A., Ahmad I., Zhao T., Aldaghri O., Eisa M. (2023). Graphene in Polymeric Nanocomposite Membranes—Current State and Progress. Processes.

[79] Kausar A., Ahmad I., Zhao T., Aldaghri O., Eisa M. (2023). Polymer/Graphene Nanocomposites via 3D and 4D Printing—Design and Technical Potential. Processes.

[80] Xu F., Gao M., Wang H., Liu H., Yan F., Zhao H., Yao Q. (2023). Polymer-based graphene composite molding: A review. RSC Adv..

[81] Zhu X., Yu S., Xu K., Zhang Y., Zhang L., Lou G., Wu Y., Zhu E., Chen H., Shen Z. (2018). Sustainable activated carbons from dead ginkgo leaves for supercapacitor electrode active materials. Chem. Eng. Sci..

[82] Zhang J., Zhao X. (2012). Conducting polymers directly coated on reduced graphene oxide sheets as high-performance supercapacitor electrodes. J. Phys. Chem. C.

[83] Chandra S., Patel M.D., Lang H., Bahadur D. (2015). Dendrimer-functionalized magnetic nanoparticles: A new electrode material for electrochemical energy storage devices. J. Power Sources.

[84] Zhang L.L., Zhao X. (2009). Carbon-based materials as supercapacitor electrodes. Chem. Soc. Rev..

[85] Selvaganesh S.V., Mathiyarasu J., Phani K., Yegnaraman V. (2007). Chemical synthesis of PEDOT–Au nanocomposite. Nanoscale Res. Lett..

[86] Ezeigwe E.R., Tan M.T., Khiew P.S., Siong C.W. (2015). One-step green synthesis of graphene/ZnO nanocomposites for electrochemical capacitors. Ceram. Int..

[87] Nayak A.K., Das A.K., Pradhan D. (2017). High performance solid-state asymmetric supercapacitor using green synthesized graphene–WO3 nanowires nanocomposite. Acs Sustain. Chem. Eng..

[88] Çıplak Z., Yıldız A., Yıldız N. (2020). Green preparation of ternary reduced graphene oxide-au@ polyaniline nanocomposite for supercapacitor application. J. Energy Storage.

[89] Arthisree D., Madhuri W. (2020). Optically active polymer nanocomposite composed of polyaniline, polyacrylonitrile and green-synthesized graphene quantum dot for supercapacitor application. Int. J. Hydrog. Energy.

[90] Sumboja A., Wang X., Yan J., Lee P.S. (2012). Nanoarchitectured current collector for high rate capability of polyaniline based supercapacitor electrode. Electrochim. Acta.

[91] Chakraborty S., Mary N. (2020). Biocompatible supercapacitor electrodes using green synthesised ZnO/Polymer nanocomposites for efficient energy storage applications. J. Energy Storage.

[92] Burkhardt S.E., Bois J., Tarascon J.-M., Hennig R.G., Abruña H.D. (2013). Li-carboxylate anode structure-property relationships from molecular modeling. Chem. Mater..

[93] Xu W., Read A., Koech P.K., Hu D., Wang C., Xiao J., Padmaperuma A.B., Graff G.L., Liu J., Zhang J.-G. (2012). Factors affecting the battery performance of anthraquinone-based organic cathode materials. J. Mater. Chem..

[94] Zou C., Zhang L., Hu X., Wang Z., Wik T., Pecht M. (2018). A review of fractional-order techniques applied to lithium-ion batteries, lead-acid batteries, and supercapacitors. J. Power Sources.

[95] Luo Y., Guo R., Li T., Li F., Liu Z., Zheng M., Wang B., Yang Z., Luo H., Wan Y. (2019). Application of Polyaniline for Li-Ion Batteries, Lithium–Sulfur Batteries, and Supercapacitors. ChemSusChem.

[96] Wu B., Ren Y., Li N., Soylu S. (2011). LiFePO_4_ Cathode Material. Electric Vehicles.

[97] Thackeray M.M., Wolverton C., Isaacs E.D. (2012). Electrical energy storage for transportation—approaching the limits of, and going beyond, lithium-ion batteries. Energy Environ. Sci..

[98] Liu Y., Zhang N., Jiao L., Chen J. (2015). Tin nanodots encapsulated in porous nitrogen-doped carbon nanofibers as a free-standing anode for advanced sodium-ion batteries. Adv. Mater..

[99] Li S., Luo Y., Lv W., Yu W., Wu S., Hou P., Yang Q., Meng Q., Liu C., Cheng H.M. (2011). Vertically Aligned Carbon Nanotubes Grown on Graphene Paper as Electrodes in Lithium-Ion Batteries and Dye-Sensitized Solar Cells. Adv. Energy Mater..

[100] Chang C.-H., Manthiram A. (2017). Covalently grafted polysulfur–graphene nanocomposites for ultrahigh sulfur-loading lithium–polysulfur batteries. ACS Energy Lett..

[101] Jiao X., Liu Y., Li T., Zhang C., Xu X., Kapitanova O.O., He C., Li B., Xiong S., Song J. (2019). Crumpled nitrogen-doped graphene-wrapped phosphorus composite as a promising anode for lithium-ion batteries. ACS Appl. Mater. Interfaces.

[102] Guo C.X., Wang M., Chen T., Lou X.W., Li C.M. (2011). A Hierarchically Nanostructured Composite of MnO_2_/Conjugated Polymer/Graphene for High-Performance Lithium Ion Batteries. Adv. Energy Mater..

[103] Li Z.-F., Zhang H., Liu Q., Liu Y., Stanciu L., Xie J. (2014). Novel pyrolyzed polyaniline-grafted silicon nanoparticles encapsulated in graphene sheets as Li-ion battery anodes. ACS Appl. Mater. Interfaces.

[104] Guo W., Su J., Li Y.-H., Wan L.-J., Guo Y.-G. (2012). Nitroxide radical polymer/graphene nanocomposite as an improved cathode material for rechargeable lithium batteries. Electrochim. Acta.

[105] Chae C., Kim K.W., Yun Y.J., Lee D., Moon J., Choi Y., Lee S.S., Choi S., Jeong S. (2016). Polyethylenimine-mediated electrostatic assembly of MnO_2_ nanorods on graphene oxides for use as anodes in lithium-ion batteries. ACS Appl. Mater. Interfaces.

[106] Song Z., Xu T., Gordin M.L., Jiang Y.-B., Bae I.-T., Xiao Q., Zhan H., Liu J., Wang D. (2012). Polymer–graphene nanocomposites as ultrafast-charge and-discharge cathodes for rechargeable lithium batteries. Nano Lett..

[107] Beladi-Mousavi S.M., Sadaf S., Mahmood A.M., Walder L. (2017). High Performance Poly (viologen)–Graphene Nanocomposite Battery Materials with Puff Paste Architecture. ACS Nano.

[108] Yao X., Zhao Y. (2017). Three-dimensional porous graphene networks and hybrids for lithium-ion batteries and supercapacitors. Chem.

[109] Sun W., Wang Y. (2014). Graphene-based nanocomposite anodes for lithium-ion batteries. Nanoscale.

[110] Riyanto E., Kristiantoro T., Martides E., Prawara B., Mulyadi D. (2023). Lithium-ion battery performance improvement using two-dimensional materials. Mater. Today Proc..

[111] Pourzare K., Mansourpanah Y., Farhadi S. (2016). Advanced nanocomposite membranes for fuel cell applications: A comprehensive review. Biofuel Res. J..

[112] Ozoemena K.I. (2016). Nanostructured platinum-free electrocatalysts in alkaline direct alcohol fuel cells: Catalyst design, principles and applications. RSC Adv..

[113] Rahimnejad M., Ghoreyshi A.A., Najafpour G., Jafary T. (2011). Power generation from organic substrate in batch and continuous flow microbial fuel cell operations. Appl. Energy.

[114] Ding H., Wang S., Long Y., Chan S.H. (2020). Non-aqueous solution synthesis of Pt-based nanostructures for fuel cell catalysts. Mater. Today Energy.

[115] Tan Q., Qu T., Shu C.-Y., Liu Y., He Y., Zhai W., Guo S.-W., Liu L., Liu Y.-N. (2019). High-Performance Polymer Fiber Membrane Based Direct Methanol Fuel Cell System with Non-Platinum Catalysts. ACS Sustain. Chem. Eng..

[116] Jung H.-Y., Roh S.-H. (2017). Carbon nanofiber/polypyrrole nanocomposite as anode material in microbial fuel cells. J. Nanosci. Nanotechnol..

[117] Chi M., He H., Wang H., Zhou M., Gu T. (2013). Graphite felt anode modified by electropolymerization of nano-polypyrrole to improve microbial fuel cell (MFC) production of bioelectricity. J. Microb. Biochem. Technol. S.

[118] Lv Z., Chen Y., Wei H., Li F., Hu Y., Wei C., Feng C. (2013). One-step electrosynthesis of polypyrrole/graphene oxide composites for microbial fuel cell application. Electrochim. Acta.

[119] Yuan H., Deng L., Chen Y., Yuan Y. (2016). MnO_2_/Polypyrrole/MnO_2_ multi-walled-nanotube-modified anode for high-performance microbial fuel cells. Electrochim. Acta.

[120] Xu L., Zhang G.-Q., Yuan G.-E., Liu H.-Y., Liu J.-D., Yang F.-L. (2015). Anti-fouling performance and mechanism of anthraquinone/polypyrrole composite modified membrane cathode in a novel MFC–aerobic MBR coupled system. RSC Adv..

[121] Rahman M.A., Yagyu J., Islam M.S., Fukuda M., Wakamatsu S., Tagawa R., Feng Z., Sekine Y., Ohyama J., Hayami S. (2023). Three-Dimensional Sulfonated Graphene Oxide Proton Exchange Membranes for Fuel Cells. ACS Appl. Nano Mater..

[122] Lee D., Yang H., Park S., Kim W. (2014). Nafion/graphene oxide composite membranes for low humidifying polymer electrolyte membrane fuel cell. J. Membr. Sci..

[123] Yuan X.-H., Yan G.-D., Li H.-T., Liu X., Su C.-Q., Wang Y.-P. (2022). Research on energy management strategy of fuel cell–battery–supercapacitor passenger vehicle. Energy Rep..

[124] Xue Y., Sun S., Wang Q., Dong Z., Liu Z. (2018). Transition metal oxide-based oxygen reduction reaction electrocatalysts for energy conversion systems with aqueous electrolytes. J. Mater. Chem. A.

[125] Ni J., Li Y. (2016). Carbon nanomaterials in different dimensions for electrochemical energy storage. Adv. Energy Mater..

[126] Zhong C., Deng Y., Hu W., Qiao J., Zhang L., Zhang J. (2015). A review of electrolyte materials and compositions for electrochemical supercapacitors. Chem. Soc. Rev..

[127] Wan C., Jiao Y., Liang D., Wu Y., Li J. (2018). A geologic architecture system-inspired micro-/nano-heterostructure design for high-performance energy storage. Adv. Energy Mater..

[128] Ouyang W., Sun J., Memon J., Wang C., Geng J., Huang Y. (2013). Scalable preparation of three-dimensional porous structures of reduced graphene oxide/cellulose composites and their application in supercapacitors. Carbon.

[129] Yang X., Fei B., Ma J., Liu X., Yang S., Tian G., Jiang Z. (2018). Porous nanoplatelets wrapped carbon aerogels by pyrolysis of regenerated bamboo cellulose aerogels as supercapacitor electrodes. Carbohydr. Polym..

[130] Mensah-Darkwa K., Zequine C., Kahol P.K., Gupta R.K. (2019). Supercapacitor energy storage device using biowastes: A sustainable approach to green energy. Sustainability.

[131] Holze R. (2023). Composites of Intrinsically Conducting Polymers with Carbonaceous Materials for Supercapacitors—An Update. Univers. J. Electrochem..

[132] Akhter J.S., Ahmad A., Sharma R.K., Singh R., Mohd A. (2023). Polymers/graphene derivative–based nanocomposites as electrode materials for supercapacitors. Advances in Electronic Materials for Clean Energy Conversion and Storage Applications.

[133] Wang Z., Liu L., Zhang Y., Huang Y., Liu J., Zhang X., Liu X., Teng H., Zhang X., Zhang J. (2023). A Review of Graphene-Based Materials/Polymer Composite Aerogels. Polymers.

[134] Zhang M., Wang L., Xu H., Song Y., He X. (2023). Polyimides as Promising Materials for Lithium-Ion Batteries: A Review. Nano-Micro Lett..

[135] Lv Z.-C., Wang P.-F., Wang J.-C., Tian S.-H., Yi T.-F. (2023). Key Challenges, Recent Advances and Future Perspectives of Rechargeable Lithium-Sulfur Batteries. J. Ind. Eng. Chem..

[136] Moyseowicz A., Minta D., Gryglewicz G. (2023). Conductive Polymer/Graphene-based Composites for Next Generation Energy Storage and Sensing Applications. ChemElectroChem.

[137] De Bortoli B.F., Camargo M.C.R., de Oliveira Polkowski R.D., de Albuquerque R.F.C. (2023). Graphene: An overview of technology in the electric vehicles of the future. SAE Tech. Pap..

[138] Starowicz A., Zieliński M., Rusanowska P., Dębowski M. (2023). Microbial Fuel Cell Performance Boost through the Use of Graphene and Its Modifications. Energies.

[139] Fernández-Sotillo A., Ferreira-Aparicio P. (2023). Durable corrosion-resistant coating based in graphene oxide for cost-effective fuel cells components. Iscience.

[140] Barik B., Yun Y., Kumar A., Bae H., Namgung Y., Park J.-Y., Song S.-J. (2023). Highly enhanced proton conductivity of single-step-functionalized graphene oxide/nafion electrolyte membrane towards improved hydrogen fuel cell performance. Int. J. Hydrog. Energy.

[141] Kausar A. (2019). Advances in polymer-anchored carbon nanotube foam: A review. Polym. Plast. Technol. Mater..

[142] Zargar V., Asghari M., Dashti A. (2015). A review on chitin and chitosan polymers: Structure, chemistry, solubility, derivatives, and applications. ChemBioEng Rev..

[143] Shi K., Yang X., Cranston E.D., Zhitomirsky I. (2016). Efficient lightweight supercapacitor with compression stability. Adv. Funct. Mater..

[144] Wang K., Li L., Zhang T., Liu Z. (2014). Nitrogen-doped graphene for supercapacitor with long-term electrochemical stability. Energy.

[145] Olivetti E.A., Cullen J.M. (2018). Toward a sustainable materials system. Science.

[146] Sharma R., Jafari S.M., Sharma S. (2020). Antimicrobial bio-nanocomposites and their potential applications in food packaging. Food Control.

